# Molecular Characteristic, Protein Distribution and Potential Regulation of HSP90AA1 in the Anadromous Fish *Coilia nasus*

**DOI:** 10.3390/genes7020008

**Published:** 2016-01-28

**Authors:** Di-An Fang, Jin-Rong Duan, Yan-Feng Zhou, Min-Ying Zhang, Dong-Po Xu, Kai Liu, Pao Xu

**Affiliations:** 1 Freshwater Fisheries Research Center, Chinese Academy of Fishery Sciences, Shanshui Road 9, Wuxi 214128, China; duanjr@ffrc.cn (J.-R.D.); zhouyf@ffrc.cn (Y.-F.Z.); zhangmy@ffrc.cn (M.-Y.Z.); xudp@ffrc.cn (D.-P.X.); liuk@ffrc.cn (K.L.); 2 Scientific Observing and Experimental Station of Fishery Resources and Environment in the Lower Reaches of the Yangtze River, Ministry of Agriculture, Xuejiali 69, Wuxi 214128, China

**Keywords:** *Coilia nasus*, HSP90AA1 expression, anadromous fish, migration mechanism

## Abstract

Heat shock proteins play essential roles in basic cellular events. Spawning migration is a complex process, with significant structural and biochemical changes taking place in the adult gonad. To date, the molecular mechanisms underlying migration reproductive biology remain undetermined. In this regard, a full length HSP90AA1 comprising 2608 nucleotides from the anadromous fish *Coilia nasus* was characterized, encoding 742 amino acid (aa) residues with potential phosphorylation sites. HSP90AA1 mRNA transcripts were detected in all organs, especially in the gonad. Furthermore, the greatest transcript levels were found during the developmental phase, while the lowest levels were found during the resting phase. In addition, the strongest immunolabeling positive signal was found in the primary spermatocyte and oocyte, with lower positive staining in secondary germ cells, and a weak or absent level in the mature sperm and oocyte. Interestingly, HSP90AA1 was mainly located in the cytoplasm of germ cells. These results are important for understanding the molecular mechanism of anadromous migration reproductive biology. In combination with data from other fish species, the result of this present study may facilitate further investigations on the spawning migration mechanism.

## 1. Introduction

Spawning migration is a complex process, with significant structural and biochemical changes take place in the adult gonad. *Coilia nasus* is a kind of small to moderately sized fish in Clupeiformes, Engraulidae [1,2]. It is famous for its important anadromous fishery resource, nutritive value and delicacy in Chinese fishery [3], which is widely distributed in the Yellow Sea and East Sea as well as Ariake Bay [2,4]. As an anadromous species, the spawning migration of *C. nasus* was classically considered as a one-time seasonal reproductive behavior that covers thousands of miles [5]. Mature individuals migrate upriver and spawn in the lower and middle reaches of the Yangtze River and other rivers in China [2], and then the spherical eggs float down and hatch near the river mouth [4]. Adults of *C. nasus* spend most of their lives in the marine environment [4,6]. However, excessive fishing and changes in aquatic ecology have almost led to the extinction of this species in the middle reaches of the Yangtze River [6,7]. Mechanism study of anadromous migration is identified as one of the most valid methods to address the depletion of fish germplasm resources nowadays [8,9]. Relative to mammals, *C. nasus* requires more complicated environments to induce spawning migration [2,7]. Prior molecular studies in this species were limited to studying genetic diversity [2,10,11]. However, the molecular mechanism underlying migration reproductive biology is not yet to be determined.

We recently demonstrated that the elevation of the heat shock protein (HSP) activity and resulting gonad development are important inducible events [12,13]. These results suggest an important regulation function of HSP in the migration process, although there is still little information regarding HSP changes with the onset of spawning migration. HSPs are key components in modulating stress responses, especially water temperature and flow [14]. As in mammals, the HSP90 in fish have been related to cytoprotection and cell survival [15,16], exerting a protective and inducible role [17,18]. Two HSP90 cytosolic isoforms have been reported: the HSP90-β is constitutive and mainly associated with early embryonic development and several cellular pathways [15], the HSP90-α (also named HSP90-α (cytosolic), class A member 1, HSP90AA1) is inducible and associated with stress-induced cytoprotection. Therefore, a better understanding of the unique function of HSP90AA1 will be of importance in future mechanism research on migration processes.

Anadromous fish spawning migration is a highly complex temporal event and an anti-stress process [19]. During fish spawning migration, different environmental changes such as water flow and temperature will induce numerous adaptive gene up/down regulation to encounter the migration behavior. HSP90 is essential for various cellular processes such as protein folding, protein degradation, signal transduction cascades, and morphological evolution [15]. HSP90AA1 is a major soluble protein in the cell and most commonly located in the cytoplasm [20]. A small fraction of HSP90 is also present in the nucleus where it shows several structural and functional properties [21]. HSP90 is a member of the ATPase/kinase GHKL superfamily (comprising DNA Gyrase, HSP90, Histidine kinase and MutL proteins), which is characterized by the presence of a unique ATP binding cleft [20,21]. Compared to other chaperone proteins, HSP90AA1 has a highly selective substrate recognition and generally low affinity for unfolded proteins [22]. They play essential roles in basic cellular events by assisting unfolded proteins, which gives the cell the required time to repair or re-synthesize damaged proteins [23,24]. In addition to regulating the proper folding of a given protein exposed to the environmental stress, HSP90AA1 had been proven to be related to normal protein trafficking, transcriptional regulation, and epigenetic regulation of gene expression [15,20].

In this study, we cloned the inducible *HSP90AA1* gene from the *C. nasus* gonad and investigated organ distribution and temporal mRNA expression in the gonad during the spawning migration period. Further *HSP90AA1* protein distribution was identified in the testis and ovary by immunohistochemical (IHC) and immunofluorescence (IF) methods. Insight into the *HSP90AA1* gene and its expression during spawning migration is important for understanding the molecular mechanisms of anadromous migration reproductive biology. In combination with data from other fish species, the results of this present study may facilitate further investigations on spawning migration mechanisms.

## 2. Results and Discussion

### 2.1. Characterization of HSP90AA1 cDNA

**Figure 1:**
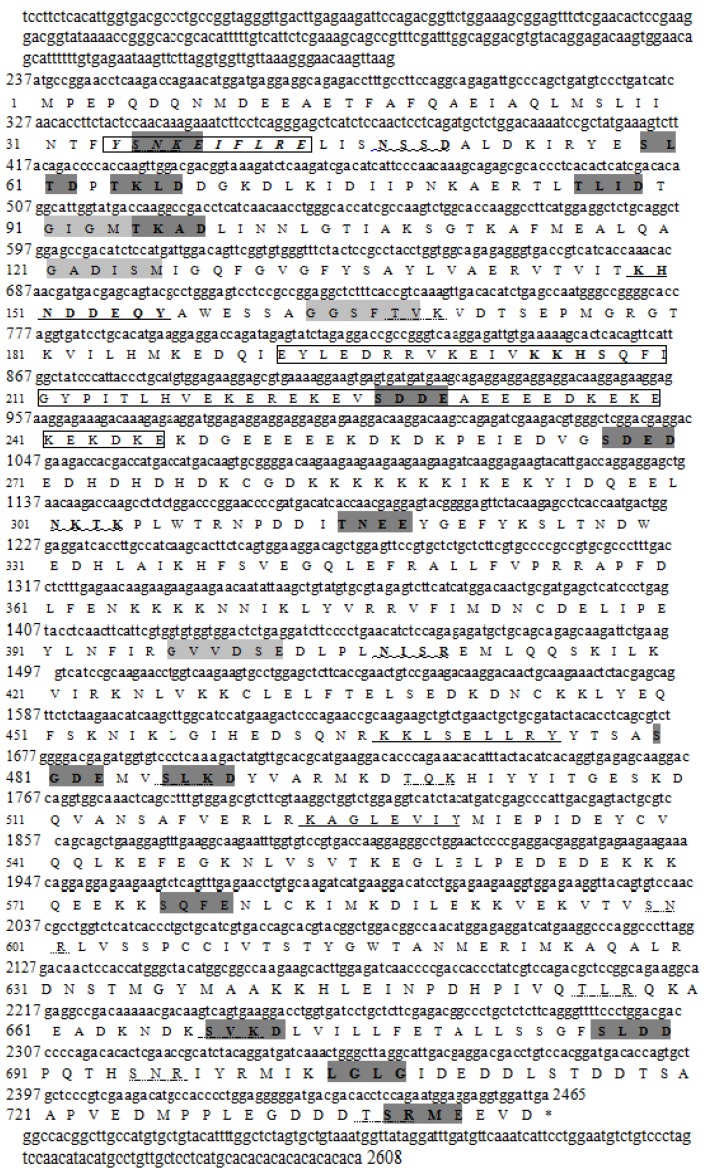
Nucleotide and deduced amino acid sequences of HSP90AA1 cDNA. The lower case letters indicate 5′ and 3′ untranslated regions and upper case letters indicate the coding region. The characteristic HSP90 family signatures are boxed. Asparagine _glycosylation sites underlined with wavy lines. PKC_phospho_site is underlined with dotted lines. Tyrosinase_phospho_site is underlined with solid lines. CK2 phospho_site is in dark grey bold and Myristyl site is in light gray. The cytoplasmic C-terminal region EEVD is shown and the stop codon (tga) is indicated with an asterisk, respectively.

Using RACE PCR method, a full-length cDNA of *HSP90AA1* (2680 bp) was cloned and sequenced, which contained a predicted open reading frame (ORF) of 2229 bp, beginning with a methionine codon at position 237 and ending with a TGA termination codon at position 2465. The 3′-untranslated region is 143 bp in length from 2466 to 2608 bp (Figure 1). The complete sequence GenBank accession number is KT387606. The encoded 742 amino acid polypeptide had a calculated molecular mass of 85.5 kDa and a predicted isoelectric point of 4.91. Using InterPro searches, two HSP90 family signature motifs were identified in the HSP90AA1 protein sequence (Figure 1). Potential phosphorylation sites were identified in HSP90AA1 using the Prosite scan by PBIL [25]. HSP90AA1 contained four Asn_glycosylation sites, two cAMP-phospho-sites (cAMP- and cGMP-dependent protein kinase phosphorylation site), 15 CK2-phospho-sites (casein kinase II phosphorylation site), four MYRISTYL sites (N-myristoylation site) and nine protein kinase C phosphorylation sites (PKC phosphorylation site), three TYR-phospho sites (tyrosine kinase phosphorylation site), a glycine-rich region profile motif (GLY-RICH) and a LYS-rich region profile motif (LYS-RICH). It is now widely accepted that some specific factors (such as temperature and water flow stress) are imprinted to *C. nasus* during upstream migration, and that these factors evoke adult *C. nasus* to recognize them during spawning and upstream migrations [5]. Several similar post-translational modifications are also found in HSP90AA1, which suggested that these structure domains may be closely related with its spawning function in different migration phases.

In the present study, analysis of HSP90AA1 revealed that it has two signature sequences motifs consistent with other HSP90 family proteins: a nonorganellar stress protein motif and an extreme C-terminal domain. This suggests that HSP90AA1 contains the typical conserved structural features of other eukaryotic cytoplasmic HSP90s. Moreover, the C-terminal MEEVD characteristic of HSP90AA1 mediate inter-domain communication and peptide-binding capacity [26], as well as other additional important residues involved in ATP hydrolysis, ATP binding, ATPase activity, and interdomain interaction. Phosphorylation by casein kinase II was also detected, which suggested that both HSP90AA1 genes are functional.

### 2.2. Expression Patterns of HSP90AA1

**Figure 2:**
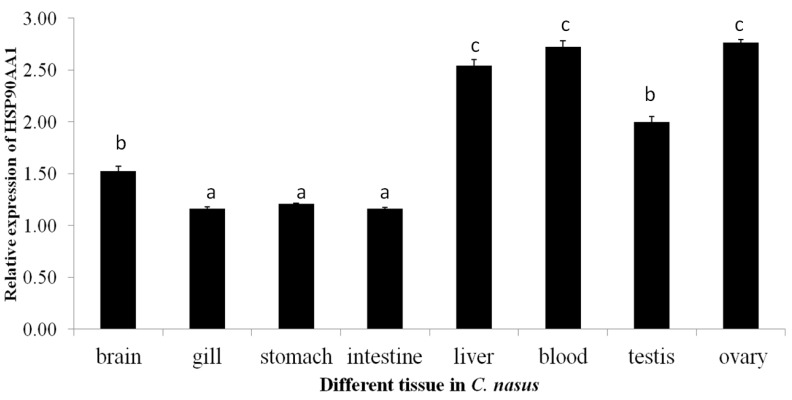
Relative expression levels of *HSP90AA1* in different tissues from *C. nasus*. Data were expressed as the mean fold difference (mean ± SE, *n* = 3). Expression values were normalized to those of 18sRNA. Values with the different superscript letters are significantly different (*p* > 0.05, a < b < c).

**Figure 3:**
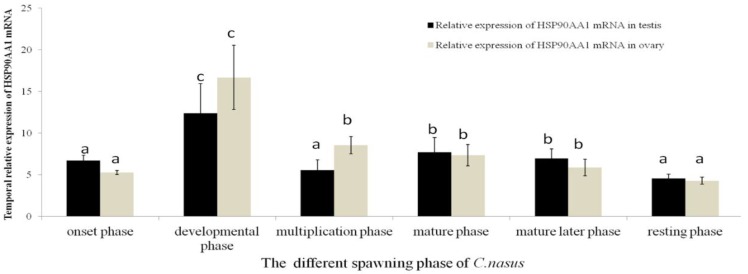
Temporal expression of *HSP90AA1* mRNA in the testis and ovary during the spawning phase. The fish different spawning phase [10,27]: onset phase (Chongming section in March), developmental phase (Nantong section in March to April), multiplication phase (Jingjiang/Zhenjiang section in April to May), mature phase (Dangtu/Anqing section in late May to early June), mature later phase (Anqing section in late June to early July), and resting phase (Anqing section in mid to late July). Expression values were normalized to those of 18sRNA. Data were expressed as the mean fold difference (mean ± SE, *n* = 3). Values with the different superscript letters are of significantly different (*p* > 0.05, a < b < c).

The mRNA transcript of *HSP90AA1* was expressed universally in all the investigated organs, including the brain, gill, stomach, intestine, liver, blood, testis and ovary. Expression was high in gonads, liver, and blood (Figure 2) with lower levels in the digestive and nervous system organs, and the lowest levels were detected in the muscle. The temporal expression of the *HSP90AA1* mRNA transcripts during the migration cycle is presented in Figure 3. During the migration cycle in the gonad, expression of *HSP90AA1* mRNA up-regulated to peak expression during the developmental phase, and high expression was maintained during the multiplication and mature period. Then, expression was down-regulated, and significantly lower expression levels were found during the resting phase (*p* < 0.05). Comparing the temporal expression between the testes with ovary, high expression levels (*p* > 0.05) were detected in the ovary. Overall, *HSP90AA1* mRNA transcripts were maintained at high levels during the migration stages. The high levels of *HSP90AA1* mRNA observed in the multiplication stage may imply that *HSP90AA1* is also an essential promoter in anadromous fish migration [27,28,29]. In our previous migration behavior study, it is found that higher temperature and water flow rate both speed up the migration reaction rate markedly [12]. The results suggested that there is a positive relationship between the Yangtze River water surface temperature and the onset timing of *C. nasus* spawning migration rate [12]. In some usual cases, fish will encounter lots of stress such as high temperatures and a rising tide. During *C. nasus* migration, it was seen that not only does the constitutive form of HSP90 accumulate, but the mRNAs coding for HSP90 proteins are also up-regulated. Therefore, a high level of *HSP90AA1* expression in the gonad might lead to an increase in the level of the protein, and that in turn could be involved in high reproductive migration efficiency. Taken together, dramatic transformations and cellular differentiation take place in all these development and migration periods, so that migration behavior is associated with high expression levels of *HSP90AA1*.

### 2.3. Localization of HSP90AA1 in Gonads

**Figure 4:**
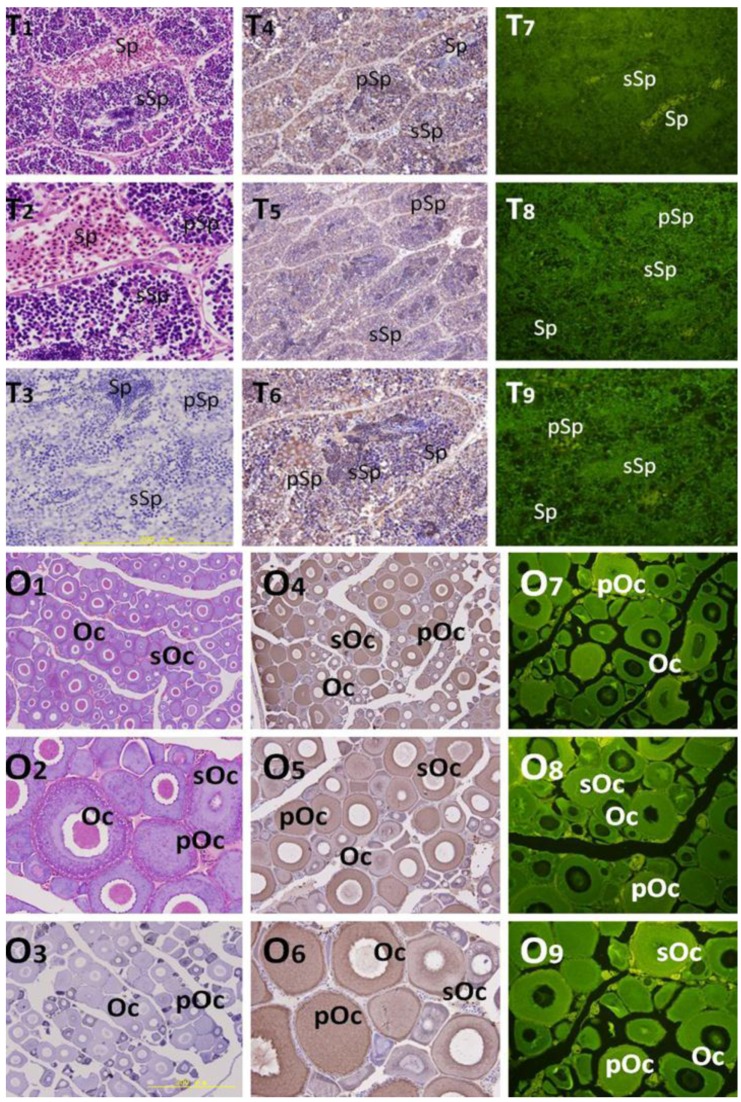
Localization of HSP90AA1 in the testis and ovary. Immunohistochemical positive signals of anti-HSP90 immunolabeling are shown in brown and immunofluorescence positive signals are shown in green. (T1–T2): whole testes section stained with hematoxylin-eosin; (T3): negative control. T4-T6 and T7-T9 are shown different part and developmental phase of testis for IHC and IF, respectively. pSp: primary spermatocytes, sSp: secondary spermatocyte, and Sp: spermatids. (O1–O2): whole testes section stained with hematoxylin-eosin; (O3): negative control. O4–O6 and O7–O9 are shown different part and developmental phase of ovary for IHC and IF, respectively. pOc: primary oocyte, sOc: secondary oocyte, and Oc: oocyte.

Localization of the HSP90AA1 protein was studied by IHC and IF. Whole sections stained with hematoxylin-eosin (H & E) and with anti-HSP90 immunolabeling (counterstained with H & E) are shown in Figure 4. Immunoreactive positive signals in brown and in green for the HSP90AA1 protein were detected for IHC and IF test, respectively. Within the testis and ovary, the strongest signals for HSP90AA1 were found in the primary spermatogonia/oocyte, with lower positive signals in the secondary spermatocyte/oocyte, and weak or absent signals in the mature sperm/oocyte. Moreover, the HSP90AA1 protein was concentrated mainly in the cytoplasm of developmental germ cells. There are no positive signals in the negative control, which was incubated with pre-immune rabbit serum (Figure 4).

HSP90AA1 is essential for various cellular processes, such as protein folding, protein degradation, signal transduction cascades, and morphological evolution [14,15]. IHC results revealed that there was higher expression level of HSP90AA1 in the early stage germ cell including the primary spermatogonia and oocyte. Weak or absent expression was seen in the mature germ cells. These results showed that the protein is abundant existing in germ cells from the beginning of the meiotic phase, which indicated that it is essential for germ proliferation and differentiation [23,30,31]. These findings are in agreement with data from other species and suggest that the HSP90AA1 protein is primarily needed during the initial steps of gametogenesis and migration behavior [28,31,32]. Moreover, the HSP90AA1 protein is found to be abundant in the cytoplasm of developmental and proliferative stage germ cells. The presence of HSP90AA1 in these stages (*i.e.*, germ cell developmental and proliferative stage) suggests that there is a very active cytoplasmic protein assembling machinery in which additional proteins needed for cell division are generated [15,33]. It has been suggested that HSP90AA1 regulates microfilament organization in a manner dependent on its phosphorylation and oligomeric status [34]. Therefore, it is possible that HSP90AA1 modulates migration in *C. nasus* by regulating cytoplasmic organization in germ cells during cell division and differentiation.

## 3. Materials and Methods

### 3.1. Fish Sampling and Organ Collection

**Figure 5:**
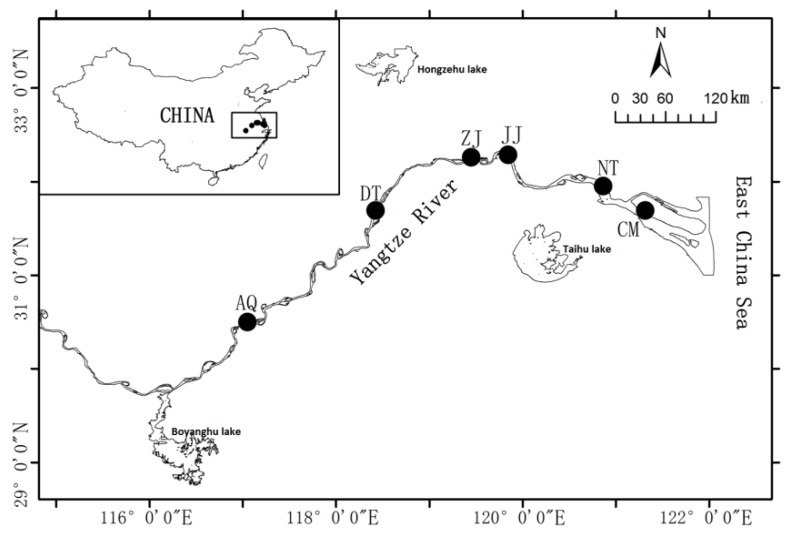
Sampling distribution in the middle and lower reach of the Yangze River. Black dot display the sampling site. AQ: Anqing; DT: Dangtu; ZJ: Zhenjiang; JJ: Jingjiang; NT: Nantong; CM: Chongming.

Drift net was used to sample the live healthy fish. Six geographical populations of *C. nasus* were collected from six major regional habitats in Yangtze River (Figure 5) during the anadromous period (from March to July, 2014). All collected individuals were caught by fisherman (total of 48 individuals with eight in each river section) and then transferred to the laboratory in dry ice boxes. Different tissues (including the brain, gill, stomach, intestine, liver, blood, testis and ovary) were removed surgically and immediately frozen in liquid nitrogen and stored at −80 °C until used. Testes were classified according to the gonad development [35,36]. All fish experimental procedures were performed in accordance with the Regulations for the Administration of Affairs Concerning Experimental Animals and fish sampling was approved and authorized by the Yangtze River Fish Committee in China.

The reproductive cycle of *C. nasus* is divided into six phases [36,37]: onset phase (Chongming section in March, Stage I), developmental phase (Nantong section in March to April, Stage II), multiplication phase (Jingjiang/Zhenjiang section in April to May, Stage III), mature phase (Dangtu/Anqing section in late May to early June, Stage IV), mature later phase (Anqing section in late June to early July, Stage V), and resting phase (Anqing section in mid to late July, Stage VI). Each reproductive phase of the fish was collected (*n* = 3, total 36 individuals) and stored at −80 °C for total RNA extraction and real-time quantitative PCR (RT-qPCR) experiment.

### 3.2. Nucleic Acid Preparation

Total RNA was extracted from different tissues using Trizol reagent (RNA Extraction kit, Invitrogen, CA, USA) according to the manufacturer’s protocol. The concentration and quality of total RNA were estimated by the spectrophotometry (absorbance at 260 nm) and agarose gel electrophoresis, respectively. Total RNA (2 μg) isolated from the gonad was reverse transcribed using the SMART™ cDNA kit (Clontech, CA, USA) for cDNA cloning, and then the PrimeScript Real-time PCR kit (TaKaRa, Toyama, Japan) was used for RT-qPCR experiments, respectively. All the primers used in this study were synthesized by Shanghai Invitrogen Biotech Co Ltd. (Invitrogen, Shanghai, China) and are listed in Table 1.

**Table 1:** Sequences of primers used in the present study.

Primer Name F—Forward/R—Reverse	DNA-Sequence 5′-3′	Annealing Temperature (°C)	Fragment Size (bp)
Gene specificity			
HSP90AA1-F1 HSP90-AA2-R1	5′-AGGAGCGTGAAAAGGAAGTGAGTGA-3′ 5′-CCTCAGAGTCCACCACACCACGAAT-3′	62.5 65.3	549
Gene-specific Primer pairs for RACE (GP)			
Gp5-1 Gp3-2	5′-GATGAGACCAGGCGGTTGGACACT-3′ 5′-CCTCAACTTCATTCGTGGTGTGGT-3′	68.5 65.5	- -
Gp5-1 Gp3-2	5′-TCATCACTCACTTCCTTTTCACGC-3′ 5′-ACCCAGAAACACATTTACTACATC-3′	66.2 67.3	- -
Primers for RT-qPCR			
HSP90AA1-F2 HSP90-AA1-R2	5′-ACCCAGAAACACATTTACTACATC-3′ 5′-GATGAGACCAGGCGGTTGGACACT-3′	62.7 61.8	330
18sRNA primers			
18sRNA-R 18sRNA-F	5′- TGATTGGGACTGGGGATTGAA-3′ 5′- TAGCGACGGGCGGTGTGT-3′	59.2 62.4	232
DNA sequencing			
T7 SP6	5′-TAATACGACTCACTATAGG-3′ 5′-ATTTAGGTGACACTATAGAA-3′	62.5 59.5	

### 3.3. Characteristics of HSP90 cDNA

Target fragments of cDNAs encoding *HSP90* were obtained from our constructed transcriptome library after using BLAST programs at the National Center for Biotechnology Information (NCBI,) [38]. To obtain the total coding sequence, rapid amplification of cDNA ends (RACE) technology was performed. The 3′ and 5′ RACE reactions were performed using the SMARTer™ RACE cDNA amplification kit (Clontech, CA, USA) according to the manufacturer’s protocol. Two pairs of gene-specific primers (Gp5-1, Gp3-1; Gp5-2, Gp3-2; Table 1) were designed basing on the obtained cDNA sequence of *HSP90AA1*. The PCR program was performed as a touch-down PCR reaction, according to the manufacturer’s protocol. The amplified cDNA fragments were cloned into the PMD18-T vector (TaKaRa, Dalian, China), and recombinants were identified by blue/white screening and confirmed by PCR. Plasmids containing the inserted *HSP90AA1* fragment were used as the template for DNA sequencing. The obtained sequences were verified and analyzed for similarity with other known *HSP90* sequences using BLAST programs at NCBI.

### 3.4. HSP90AA1 mRNA Expression Patterns and RT-qPCR Analysis

The organ-dependent *HSP90* mRNA expression was measured by RT-qPCR as the following method. Briefly, first-strand cDNA was prepared as described above (using six individuals for pooled RNA). Gene-specific primers (*HSP90AA1*-F2, *HSP90-AA1*-R2; Table 1) were designed based on the cloned *HSP90AA1* cDNA to produce an amplicon of 330 bp. All RT-qPCR reactions were performed in triplicate using extracted RNA (pooled) of the same concentration. RT-qPCR was performed in a C1000™ Thermal Cycler (BioRad CFX 96™ Real-Time System) according to the manufacturer’s instructions. The final volume of each RT-qPCR reaction was 25 μL, which contained 12.5 μL SYBR Premix ExTaq (TaKaRa, Dalian, China), 0.5 μL of diluted cDNA template, 11 μL of PCR-grade water, and 0.5 μL of each 10 μM primer. PCR conditions were as follows: 95 °C for 30 s, followed by 40 cycles of 95 °C for 5 s and a 0.5 °C/5 s incremental increase from 60 °C to 95 °C that lasted 30 s per cycle. The primers 18sRNA-R and 18sRNA-F were designed basing on the *C. nasus 18sRNA* to amplify a fragment 232 bp. Samples were run in triplicate and normalized to the selected control gene *18sRNA*. *HSP90AA1* expression levels were calculated by the 2^−ΔΔCt^ comparative CT method. Mean and standard deviations were calculated from triplicate experiments, and presented as the n-fold differences in expression relative to *18sRNA*. Data were analysized using the CFX Manager™ software version 1.6 (Bio-Rad, Hercules, CA, USA).

### 3.5. Preparation of Anti-HSP90 Antibody

The production of a synthetic peptide and monoclonal antibody was carried out commercially by Abcam (Abcam, Cambridge, UK). Briefly, a synthetic C-terminal peptide (PVEDMPPLEGDDDTSRMEEVD) for HSP90 conjugated with keyhole limpet hemocyanin was emulsified with complete (for first immunization) and incomplete (for second to fourth immunizations) Freund adjuvant, and injected into a New Zealand rabbit at intervals of 2 to 3 weeks. Before immunization and after the third and fourth injections, the rabbit was bled and serum samples were collected. An increase in antibody titers against the peptide was verified by enzyme-linked immunosorbent assay.

### 3.6. Immunohistochemistry

Six individuals (3 for male and 3 for female) were sacrificed and stored as above for IHC and IF analysis. Frozen sections were used for IHC analyses. Testis and ovary were dissected out from fish in the mature stage and fixed in 0.01 M phosphate-buffered saline (PBS) containing 4% paraformaldehyde at 4 °C for above 6 h. After washing with PBS three times, the samples were dehydrated in 30% saccharose-PBS solutions for 4 h at room temperature, and then embedded in organ optimal cutting temperature compound (Sakure, Trrance, CA, USA). Standard frozen sections of 8 μm in thickness were taken using a microtome (Leica, Bensheim, Germany). IHC was carried out essentially as described by Multhoff [33]. Briefly, after washing with 0.01 M PBS three times for 10 min each wash, sections were immersed in 0.01 M citric acid buffer (pH 6.0) containing 0.1% Tween 20, and autoclaved for 5 min. Then the sections were treated in a blocking solution (Roche, Shanghai, China), incubated with anti-HSP90 (1:200) overnight at 4 °C, and rinsed with 0.01 M PBS three times for 5 min each wash. Subsequently, the organ sections were incubated with goat anti-rabbit IgG conjugated with horseradish peroxidase for 30 min, and then rinsed with PBS three times for 5 min each wash. Immunoreactive signals were visualized using diaminobenzidine (Sigma, Shanghai, China) as the substrate. Sections were counterstained with H & E. Organ sections were also incubated with pre-immune rabbit serum and the blocking solution as the negative control.

### 3.7. Immunofluorescence

Standard frozen sections were prepared as the above IHC method. The cryosections (8 μm in thickness) were mounted on glass slides, washed in PBS, and immersed in 3% BSA for 1 h to block nonspecific binding. These slides were then incubated with primary antibodies against HSP90 at dilutions of 1:100 for 18 h at 4 °C, washed twice in PBS/Tween-20 solution, incubated with a fluorescein conjugated secondary antibody for 1 h at room temperature, and photographed with a fluorescence microscope (Leica, Bensheim, Germany). Organ sections were also incubated with PBS and the blocking solution as the negative control.

### 3.8. Statistical Analyses

Statistical analyses were performed using SPSS software version 11.0 (SPSS Inc., Chicago, IL, USA). Data are given as mean ± one standard error (SE). Statistical significances were determined by one-way ANOVA and post-hoc Duncan multiple range tests. Significance was set at *p* < 0.05 [39].

## 4. Conclusions

In conclusion, in *C. nasus* migration, the *HSP90AA1* mRNA transcript levels changed during the migration process, which was in accordance with the immunoreactive signals of HSP90AA1 in the gonads. These observations suggest that HSP90AA1 expression patterns might be closely related to migration efficiency and quantity. In the present study, the result provides an initial step towards understanding reproductive migration in anadromous fish, which will provide insight into reproductive migration mechanisms.
